# Heat activation desensitizes *Aedes aegypti* transient receptor potential ankyrin 1 (AaTRPA1) to chemical agonists that repel mosquitoes

**DOI:** 10.1016/j.pestbp.2025.106326

**Published:** 2025-02-11

**Authors:** Yeaeun Park, Peter M. Piermarini

**Affiliations:** Department of Entomology, College of Food, Agriculture and Environmental Sciences, The Ohio State University, Wooster, OH 44691, USA

**Keywords:** Electrophysiology, Mosquito, TRPA1, Catnip oil, Citronellal, Repellency

## Abstract

Mosquito transient receptor potential ankyrin 1 (TRPA1) channels are nociceptors that can be activated by noxious heat and/or chemicals (e.g., electrophiles). When activated, TRPA1 channels typically trigger avoidance behaviors. Previous studies have found that mosquito TRPA1 channels play important roles in host-seeking, preferred temperature selection, and avoidance of noxious heat and chemicals in the environment. Accordingly, TRPA1 channels are considered valuable biochemical targets for developing mosquito repellents and/or antifeedants. However, it is presently unknown whether heat activation of mosquito TRPA1 channels impacts their activation by chemical agonists that repel mosquitoes, such as catnip oil and citronellal. To address this gap in knowledge, we heterologously expressed *Aedes aegypti* TRPA1 (AaTRPA1) in *Xenopus laevis* oocytes and evaluated its electrophysiological responses to chemical agonists in the presence or absence of a heat stimulus. We found that when AaTRPA1 was heat activated it exhibited dampened electrophysiological responses to either catnip oil or citronellal. Subsequent airborne repellency bioassays with adult female *Ae. aegypti* revealed that mosquitoes were less repelled by either catnip oil or citronellal when exposed to an increase of ambient temperature that exceeded the heat activation threshold for AaTRPA1 (i.e., >32 °C); in contrast, the repellency of DEET (a non-TRPA1 agonist) was unaffected. Our results suggest that TRPA1-agonizing repellents may offer less protection from mosquitoes when ambient temperatures exceed the thermal activation threshold of mosquito TRPA1 channels. This may have important implications for the choice of mosquito repellents used during extreme heat events, which are becoming more common because of global climate change.

## 1. Introduction

TRPA1 channels are temperature/ligand-gated ion channels that can detect both noxious temperatures and/or chemicals in the environment. TRPA1 channels are widely conserved among both vertebrates and invertebrates, but their specific functions, thermal activation thresholds, and sensitivity to chemical stimuli are species-specific (Gracheva et al., 2010; Laursen et al., 2015, 2023). TRPA1 channels are typically considered nociceptors that when activated stimulate avoidance behaviors in animals (Chen and Hackos, 2015; Zhang et al., 2023). However, in some predatory/parasitic species (e.g., pit vipers, ticks, mosquitoes) they are also known to be involved in detection of infrared radiation and attraction to prey/hosts (Gracheva et al., 2010; Mitchell et al., 2017; Chandel et al., 2024).

In hematophagous adult female mosquitoes, TRPA1 was originally hypothesized to play a direct role in host seeking by functioning as a close-range peripheral heat sensor. That is, TRPA1 mRNA and/or protein expression was detected in antennae, maxillary palps, and proboscis of adult female *Anopheles gambiae* and/or *Anopheles stephensi* (Maekawa et al., 2011; Wang et al., 2009). Moreover, in adult female *An. stephensi*, surgical ablation of antennae, proboscis, or maxillary palps, or exposure to a chemical agonist of TRPA1 channels (i.e., allyl isothiocyanate), disrupted mosquito host-seeking behavior (Maekawa et al., 2011). Furthermore, when heterologously expressed in *Xenopus laevis* oocytes, *An. gambiae* TRPA1 was activated by increases of temperature from ~22 °C to ~40 °C, with maximal responses occurring near mammalian host body temperatures (Hamada et al., 2008; Wang et al., 2009). Support for this hypothesis has been bolstered by a recent study in *Ae. aegypti*, which demonstrated that TRPA1 mRNA was expressed in the distal tips of antennae and that detection of infrared radiation was disrupted by either removal of the distal ends of antennae or genetic ablation of TRPA1 (Chandel et al., 2024). TRPA1 also plays an indirect role in host seeking by steering mosquitoes away from objects that far exceed typical host body temperatures and are potentially noxious (Corfas and Vosshall, 2015).

In addition, a recent study by Li et al. (2019) has suggested that mosquito TRPA1 plays a role in preferred temperature selection by contributing to the avoidance of non-ideal high temperatures. That is, the thermal activation thresholds of heterologously expressed mosquito TRPA1 channels from temperate (*Culex pipiens*) and tropical (e.g., *Ae. aegypti, An. gambiae*) species correlated with their respective thermal niches (Li et al., 2019). For at least *Cx. pipiens* and *Ae. aegypti*, the different thermal activation thresholds in vitro correlated with behavioral differences in vivo. That is, adult female *Cx. pipiens* avoided 30 °C, a temperature above this species’ TRPA1 heat activation threshold of 21.8 °C, whereas *Ae. aegypti* did not avoid 30 °C, a temperature below this species’ heat TRPA1 threshold of 32 °C (Li et al., 2019).

Mosquito TRPA1 channels also contribute to the avoidance of potentially noxious chemicals in the environment (e.g., electrophiles), including certain natural product pesticides. For example, heterologous expression of various mosquito TRPA1 channels have shown that they are activated by a variety of plant secondary metabolites known to exhibit repellent and/or antifeedant activity against mosquitoes, including citronellal, cinnamodial, and nepetalactone, which are major constituents of citronella oil, *Cinnamosma fragrans* bark extracts, and catnip oil, respectively (Inocente et al., 2018; Kang et al., 2010; Kwon et al., 2010; Li et al., 2019; Melo et al., 2021). Moreover, genetically-modified *Ae. aegypti* lacking TRPA1 show weaker aversive responses to at least cinnamodial, nepetalactone, and catnip oil, confirming TRPA1 agonism as a component of their repellent or antifeedant mode of action in vivo (Inocente et al., 2018; Melo et al., 2021). Thus, TRPA1 channels are considered valuable biochemical targets for repellent development (Salgado, 2017).

Despite the activation of some TRPA1 channels by both noxious heat and chemicals, few studies have examined the potential interactions between these factors. In other words, does an increase of ambient temperature above the heat activation threshold for TRPA1 impact its response to a chemical agonist? This question is important to address because extreme heat events where ambient temperatures are likely to exceed 32 °C for one or multiple days, which would surpass the heat activation thresholds for all mosquito TRPA1 channels characterized to date (Li et al., 2019), are becoming more common as global climate changes (IPCC, 2023). Thus, as mosquitoes are exposed to ambient temperatures that exceed the heat activation threshold for TRPA1, it is unknown how this may impact the efficacy of certain repellents that target TRPA1, such as catnip oil and citronellal. A previous study found that heat activation of heterologously expressed mammalian TRPA1 channels desensitized them to chemical agonists (Wang et al., 2012). Whether a similar phenomenon occurs for mosquito TRPA1 channels is unknown, but previous studies have found that prolonged exposure of insect TRPA1 channels to temperatures above their thermal activation threshold can induce a desensitizing effect to heat (Kohno et al., 2010; Peng et al., 2016; Sato et al., 2014; Viswanath et al., 2003; Wang et al., 2009).

The goal of the present study was to address this gap of knowledge and test the hypothesis that heat activation of AaTRPA1 affects its activation by repellent chemical agonists. Based on the aforementioned study of mammalian TRPA1 (Wang et al., 2012), we predicted that heat activation of AaTRPA1 would desensitize it to chemical TRPA1 agonists and mosquitoes exposed to temperatures above the heat-activation threshold of AaTRPA1 would be less repelled by these agonists.

## 2. Materials and methods

### 2.1. Heterologous expression and two-electrode voltage clamping in Xenopus laevis oocytes

An *AaTrpa1* cDNA template (GenBank Accession #LC438794.1; AaTRPA1C in Li et al., 2019) was synthesized de novo (Genewiz Azenta Life Sciences, South Plainfield, NJ, USA) and sub-cloned into a pGH19 expression plasmid (Trudeau et al., 1995). Previous studies have shown that the encoded AaTRPA1 protein derived from this cDNA can be activated by either heat (>32 °C) or chemical agonists (Li et al., 2019; Melo et al., 2021). The plasmid containing the *AaTrpA1* cDNA was linearized with *NotI* restriction enzyme (Thermo Fisher Scientific, Waltham, MA, USA) and used as a template to synthesize *AaTrpa1* cRNA in vitro with a mMessage mMachine T7 Transcription kit (Thermo Fisher Scientific).

Defolliculated *Xenopus laevis* oocytes were purchased from Ecocyte Bioscience (Austin, TX, USA). Each oocyte was injected with 25 nL of *AaTrpA1* cRNA (1 ng/nL) or nuclease-free H_2_O (controls); injected oocytes were stored in sterile OR3 culture media (Romero et al., 1998) at 18 °C for 2–3 days prior to experiments. The OR3 media consisted of one pack of powdered Leibovitz L-15 media with l-glutamine (Gibco, Thermo Fisher Scientific) dissolved in ~1.8 L of deionized H_2_O (Millipore) supplemented with 1000 U penicillin-streptomycin (Gibco, Thermo Fisher Scientific) and 5 mM HEPES (pH 7.5 titrated with 1 N NaOH). The osmolality of OR3 was confirmed to be 195 ± 5 mOsmol/kg using a vapor pressure osmometer (model 5600, Wescor, Logan, UT, USA).

Two-electrode voltage clamping experiments were performed on oocytes 2–3 days after cRNA injection. The whole-cell membrane currents (I_m_) of *X. laevis* oocytes were recorded with an OC-725 oocyte clamp (Warner Instruments, Hamden, CT, USA) following a protocol similar to Inocente et al. (2018). The current injected into the cell was recorded with pCLAMP software (Clampex module, Version 10, Molecular Devices, San Jose, CA, USA) that interfaced with the oocyte clamp via a digital recording device (Digidata, Molecular Devices). The extracellular bath solution (ND96) for the oocytes consisted of 96 mM NaCl, 2 mM KCl, 1.8 mM CaCl_2_, 1.0 mM MgCl_2_, and 5 mM HEPES; the pH was adjusted to 7.5 dropwise using 1 M *N*-methyl D-glucamine. The osmolality of ND96 was confirmed to be 195 ± 5 mOsmol/kg using a vapor pressure osmometer (model 5600, Wescor). Each oocyte was placed in a bath chamber (RC-1Z, Warner Instruments) and superfused with gravity-fed ND96 at a constant flow rate (3 mL/min). Two glass microelectrodes (0.5–1.0 MΩ resistance) were filled with 3 M KCl and mounted on micromanipulators to measure the oocyte membrane voltage (V_m_) and I_m_, respectively. Once the oocyte was impaled with both electrodes and V_m_ reached a steady state, the V_m_ was clamped at a hyperpolarizing voltage (−30 mV relative to the resting V_m_) to promote inward currents upon TRPA1 activation (Inocente et al., 2018).

For chemical TRPA1 agonists, 6.2 M (96 %) citronellal (Thermo Fisher Scientific) was initially diluted to 1 M using 100 % dimethyl sulfoxide (DMSO) and then diluted to a final concentration of 1 mM (0.1 % DMSO) using ND96 on the day of experiment. In a previous study (Li et al., 2019), this concentration of citronellal elicited maximal electrophysiological responses of AaTRPA1. Catnip oil (Salvia, Delhi, India) was directly diluted to a final concentration of 0.1 % using ND96. A previous study (Melo et al., 2021) found that this concentration of catnip oil agonized AaTRPA1, albeit the oil used was from a different supplier than that used in the present study. In preliminary experiments, we confirmed that neither agonist produced inward currents in H_2_O-injected oocytes. Moreover, we confirmed that both AaTRPA1 and H_2_O-injected oocytes showed nominal responses (< 0.025 μA of inward current on average) when exposed to 0.1 % DMSO alone, the vehicle for citronellal. For all experiments, the chemical agonists were introduced to the chamber when needed using an eight-way rotary valve (model 5012, Rheodyne, Rohnert Park, CA, USA).

To increase the temperature of the oocyte bath from 23 °C to ~38 °C, an in-line solution heater and TC-324C single channel temperature controller were used (Warner Instruments). The in-line heater was connected to a two-way rotary valve downstream of the eight-way rotary valve, which allowed for rapid heating or cooling of the bath with or without chemical agonists. To record the bath temperature, a thermistor (Warner Instruments) was added to the bath immediately downstream of the oocyte and interfaced with the temperature controller.

Each oocyte was treated with only one of the chemical agonists using only one of the following 3 experimental designs. In the first design, the chemical agonist was introduced while the oocyte was exposed to ~38 °C. That is, the bath temperature was increased from 23 °C to ~38 °C within approximately 30–40 s to thermally activate AaTRPA1. The temperature was then maintained at ~38 °C for approximately 2–3 min before adding a chemical agonist (0.1 % catnip oil or 1 mM citronellal) to the bath for 40 s and then returning to ND96 to wash out the chemical agonist. The second design was very similar to the first, but the chemical agonist was added within only 30 s of thermally activating AaTRPA1. Controls for these two designs consisted of separate AaTRPA1-expressing oocytes exposed to ND96 and chemical agonists for the same periods of time, but the bath temperature remained at 23 °C.

In the third design, the chemical agonist was introduced after the oocyte was exposed to a transient increase of temperature to ~38 °C. That is, the bath temperature was increased from 23 °C to ~38 °C over 40 s to thermally activate the TRPA1 channel, but then the temperature of the bath was returned to 23 °C. Approximately 2–3 min later, a chemical agonist was added to the bath for 40 s and washed out as described above. Controls were performed as described above for the first two designs.

For each experimental design, the responses (ΔI_m_) of AaTRPA1 oocytes to chemical agonists were quantified using the Clampfit module of pCLAMP software. The ΔI_m_ was determined by subtracting the baseline I_m_ (i.e., I_m_ immediately before the application of agonist) from the maximal agonist-induced I_m_.

### 2.2. Mosquito cultures

Eggs of the Liverpool (LVP) strain of *Ae. aegypti* were obtained from the MR4 as part of BEI Resources Repository, NIAID, NIH (LVP-IB12, MRA-735, contributed by M.Q. Benedict). The eggs were reared to adults at 28 °C (80 % relative humidity; 12 h:12 h light:dark regime) as described previously (Li and Piermarini, 2024). Adults were fed with 10 % sucrose ad libitum. When additional eggs were needed, mosquitoes were fed defibrinated rabbit blood (Hemostat Labs, Dixon, CA) using a membrane feeding system (Hemotek, Blackburn, UK).

### 2.3. Mosquito airborne repellency bioassay

The airborne repellency bioassay used in the present study was modified from pre-existing choice-based bioassays (Jiang et al., 2019; Kwon et al., 2010) by adding a trap that prevented mosquitoes from escaping after making a choice and reducing the assay time from 30–60 min to 10 min. These modifications were made to more effectively expose mosquitoes to a heat treatment and reduce the likelihood of mosquitoes becoming acclimated/desensitized to, and/or intoxicated by, the repellents. In brief, two 50 mL-polypropylene centrifuge tubes (VWR International, Radnor, PA, USA) were each cut twice at 30 mL and 10 mL graduations using a heated knife, resulting in a small conical tip (i.e., from the 10 mL graduation to the tip), a relatively long cylinder (from the 30 mL to 50 mL graduations) with the removable cap, and a short cylinder (from the 30 mL to 10 mL graduations) from each tube; the short cylinders were discarded. The conical tips were carefully cut at their ends with a heated knife to produce a funnel with an opening of ~0.5 cm in diameter. Each funnel was attached to the ends of a 12.5 cm glass cylinder tube (TriKinetics Inc., Waltham, MA, USA).

Ten adult female *Ae. aegypti* (4–12 days post-eclosion) were transferred into the glass tube using a mouth aspirator and then 1000 μL pipette tips were inserted into the funnel openings to prevent mosquitoes from escaping. The glass tube containing the mosquitoes was then placed in a walk-in environmental chamber (26 °C; 70–80 % RH) for 10 min to acclimate the mosquitoes. Meanwhile, grade 1 circular filter papers (2.5 cm-diameter; Whatman, Maidstone, UK) were each placed inside the plastic caps associated with the long cylinder. One filter paper (control) received 50 μL of 100 % acetone, whereas the other filter paper (treatment) received 50 μL of 1.0 % catnip oil (0.5 mg), 2.0 % citronellal (1.0 mg), or 1.0 % DEET (0.5 mg) dissolved in 100 % acetone. These concentrations (amounts) were used because in preliminary experiments they repelled mosquitoes without causing vapor toxicity. In some experiments (negative controls), acetone was placed on both filter papers. After allowing 10 min for acetone to evaporate, the filter papers in the caps were covered with a small piece of white mesh fabric and the caps were secured to their corresponding polypropylene cylinder.

The glass tube with the mosquitoes was placed on a mug warmer (model ZJZ-KF-01, Ikago, China; ~81 cm^2^ heating surface) to heat the glass tube from 26 °C to a temperature above 32 °C, the heat activation threshold of AaTRPA1 (Li et al., 2019). For controls, the mug warmer remained at 26 °C throughout the experiment. The pipette tips were removed from the funnels and the long polypropylene cylinders (with attached caps containing a repellent or acetone) were fastened to each end of the glass tube with masking tape as shown in Fig. S1. After 10 min, the numbers of mosquitoes that entered each trap were counted and an avoidance index (AI) was calculated (Eq. 1). The AI values of −1, 0, and 1 corresponded to complete attraction, indifference, and complete repellency, respectively, to the chemical treatment (i.e., catnip oil, citronellal, or DEET). If less than 5 mosquitoes were in both traps combined, then the trial was discarded and not used for analysis.

Calculation of avoidance index (AI)

(1) $$\text{AI}=\frac{M_{a}-M_{r}}{M_{a}+M_{r}}$$

*M*_*a*_: Number of mosquitoes in the acetone-treated trap

*M*_*r*_: Number of mosquitoes in the repellent-treated trap.

To characterize how the interior temperature of the glass tube increased during these experiments, we performed mock trials (without mosquitoes) where a probe sensor thermometer (Traceable, Thermo Fisher Scientific) was positioned centrally in the glass tube. As shown in Fig. S2, the temperature inside the tube increased at a rate of ~6 °C per min and exceeded the AaTRPA1 heat activation threshold within 1 min (dashed red lines in Fig. S2) before plateauing at ~55 °C around 4 min. In preliminary trials, we observed that 80 % of the mosquitoes within the glass tube entered one of the traps within 2.4 ± 0.4 min (*n* = 15 replicates) of heat exposure (dashed magenta lines in Fig. S2). Thus, the majority of mosquitoes left the glass tube before it reached ~43 °C (Fig. S2).

### 2.4. Statistical analyses

Statistical analyses were conducted using R Studio (PBC, Boston, MA, USA) and GraphPad Prism (version 10, GraphPad, San Diego, CA, USA). All data were tested for normality using a Shapiro-Wilk test. If they were not normally distributed, then they were log transformed; any log transformed data were confirmed to be normally distributed by a Shapiro-Wilk test. To determine whether mean ΔI_m_ values produced by a chemical agonist were affected by continuous or prior heat activation of AaTRPA1, we used unpaired *t*-tests. For the mosquito airborne repellency trap bioassay, the mean AI values between the control and heat treatment were compared with unpaired *t*-tests. The results were considered statistically significant if the *p*-values were less than 0.05. Data are presented as means ± standard errors of the mean (SEM) if not stated otherwise.

## 3. Results

### 3.1. AaTRPA1 responses to chemical agonists are dampened by continuous heat activation

**Fig. 1.**
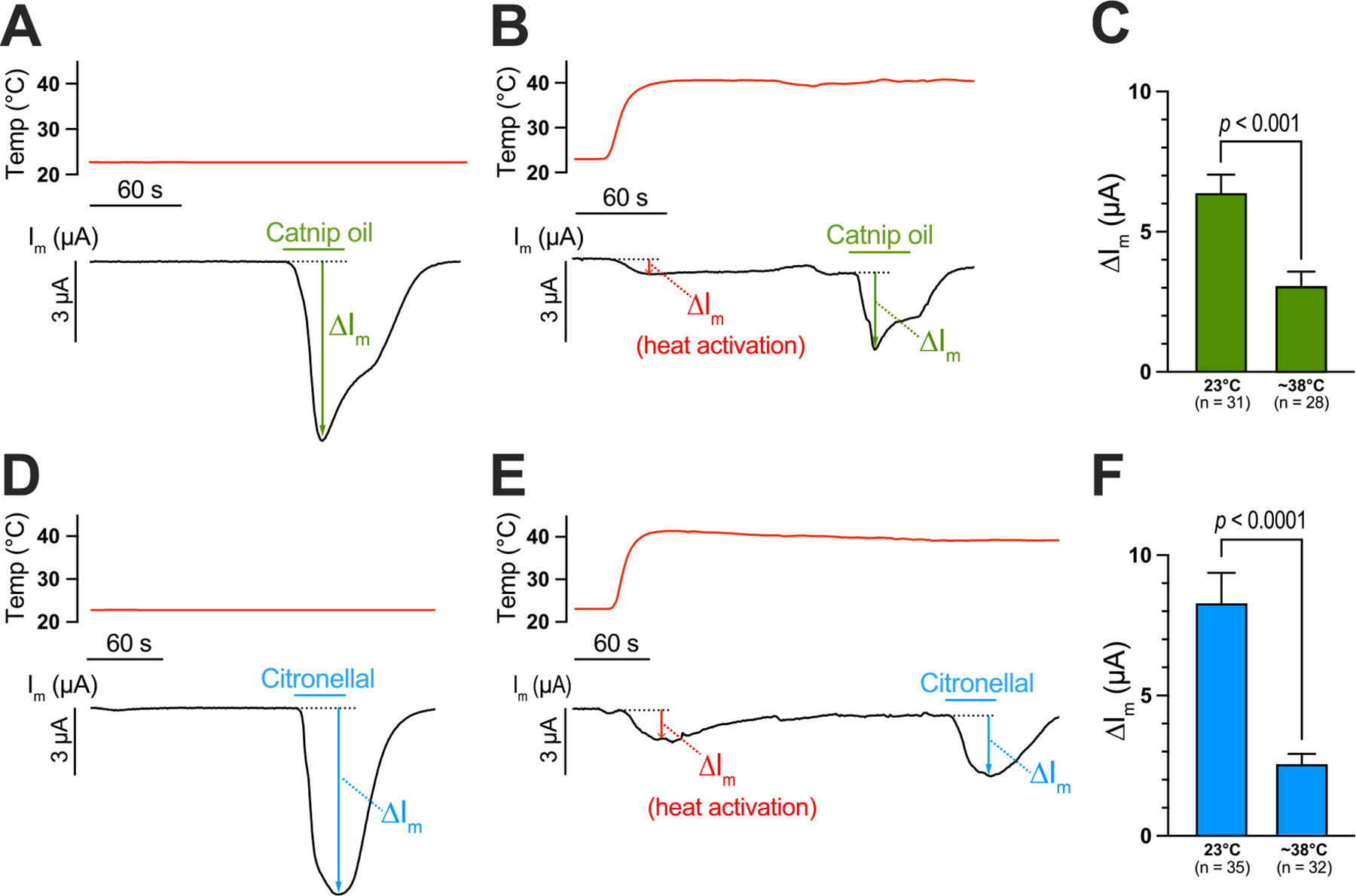
Chemical activation of AaTRPA1 is dampened by continuous heat activation. Representative traces of bath temperature (red) and I_m_ (black) in AaTRPA1 oocytes exposed to either 0.1 % catnip oil (A&B) or 1 mM citronellal (D&E) at 23 °C (A&D) or ~38 °C (B&E). Green and blue horizontal bars indicate exposure of oocytes to catnip oil or citronellal, respectively. The maximal electrophysiological responses (ΔI_m_) evoked by temperature (red), catnip oil (green), and citronellal (blue) are indicated. Panels C & F compare the mean ΔI_m_ for each agonist (catnip oil in C, citronellal in F) at 23 °C vs. ~38 °C. Values are means ± SEM. The *p*-values were determined by unpaired *t*-tests on log-transformed data.

As shown by the representative tracings in Fig. 1A&D, treatment of AaTRPA1 oocytes with catnip oil or citronellal at 23 °C, a temperature below the heat activation threshold of 32 °C for AaTRPA1 (Li et al., 2019), elicited robust inward currents of several μA (see ‘ΔI_m_’ in Fig. 1A&D). The chemical activation was reversible within a few min of washing out the agonist (Fig. 1A&D). In parallel, and in separate oocytes, we assessed the effects of continuous heat activation (first experimental design in section 2.1) on the responses of AaTRPA1 to the same chemical agonists (Fig. 1B&E). Elevating the bath temperature from 23 °C to ~38 °C, a temperature above the thermal activation threshold of AaTRPA1 (Li et al., 2019), elicited a ΔI_m_ of ~0.5–1 μA (see ‘heat activation’ in Fig. 1B, E) that slowly and partially returned towards the original baseline before stabilizing over the next few min. The ‘new’ baseline at ~38 °C was shifted on average ~0.5 μA more negative than the original baseline at 23 °C, but ~0.8 μA less negative than the ΔI_m_ associated with initial heat exposure (Fig. S3), suggesting AaTRPA1 remained active throughout the continuous heat exposure, albeit to a lesser degree than the initial heat exposure. Treatment of heat-activated oocytes at ~38 °C with catnip oil or citronellal elicited dampened ΔI_m_ responses (Fig. 1B&E) relative to those at 23 °C (Fig. 1 A&D). The chemical activation at ~38 °C was reversible (Fig. 1B&E). As shown in Fig. 1C, the mean ΔI_m_ in AaTRPA1 oocytes exposed to catnip oil at 23 °C (6.41 μA ± 0.63 μA) was ~2-fold larger than that in heat-activated AaTRPA1 oocytes exposed to catnip oil at ~38 °C (3.10 μA ± 0.48 μA). Similarly, the mean ΔI_m_ in AaTRPA1 oocytes exposed to citronellal at 23 °C (8.42 μA ± 1.04 μA) was ~3.3-times larger than that in heat-activated AaTRPA1 oocytes exposed to citronellal at ~38 °C (2.53 μA ± 0.34 μA) (Fig. 1F).

Control oocytes injected with H_2_O exhibited ΔI_m_ responses associated with the continuous temperature increase (Fig. S4A&B) that were over 90 % smaller (unpaired *t*-test, *P* < 0.0001) than those observed in AaTRPA1 oocytes (H_2_O-injected ΔI_m_ = 0.10 μA ± 0.02 μA, *n* = 10; AaTRPA1 ΔI_m_ = 1.31 μA ± 0.19 μA, *n* = 60). Neither catnip oil nor citronellal elicited detectable responses in H_2_O-injected oocytes exposed to continuous heat (Fig. S4A&B).

To determine if the chemical activation of AaTRPA1 was dampened by a shorter duration of heat activation (i.e., 30 s vs. 2–3 min), we performed experiments where the oocyte was treated with a chemical agonist within 30 s of the bath temperature increase (second experimental design in section 2.1). As shown in Fig. S5, the ΔI_m_ values in AaTRPA1 oocytes exposed to either catnip oil or citronellal at 23 °C were respectively ~4-times or 5-times larger than those in AaTRPA1 oocytes that were heat activated for only ~30 s. Thus, a prolonged heat activation was not necessary to dampen the chemical activation of AaTRPA1.

### 3.2. AaTRPA1 responses to chemical agonists are unaffected by a prior, brief heat activation

**Fig. 2.**
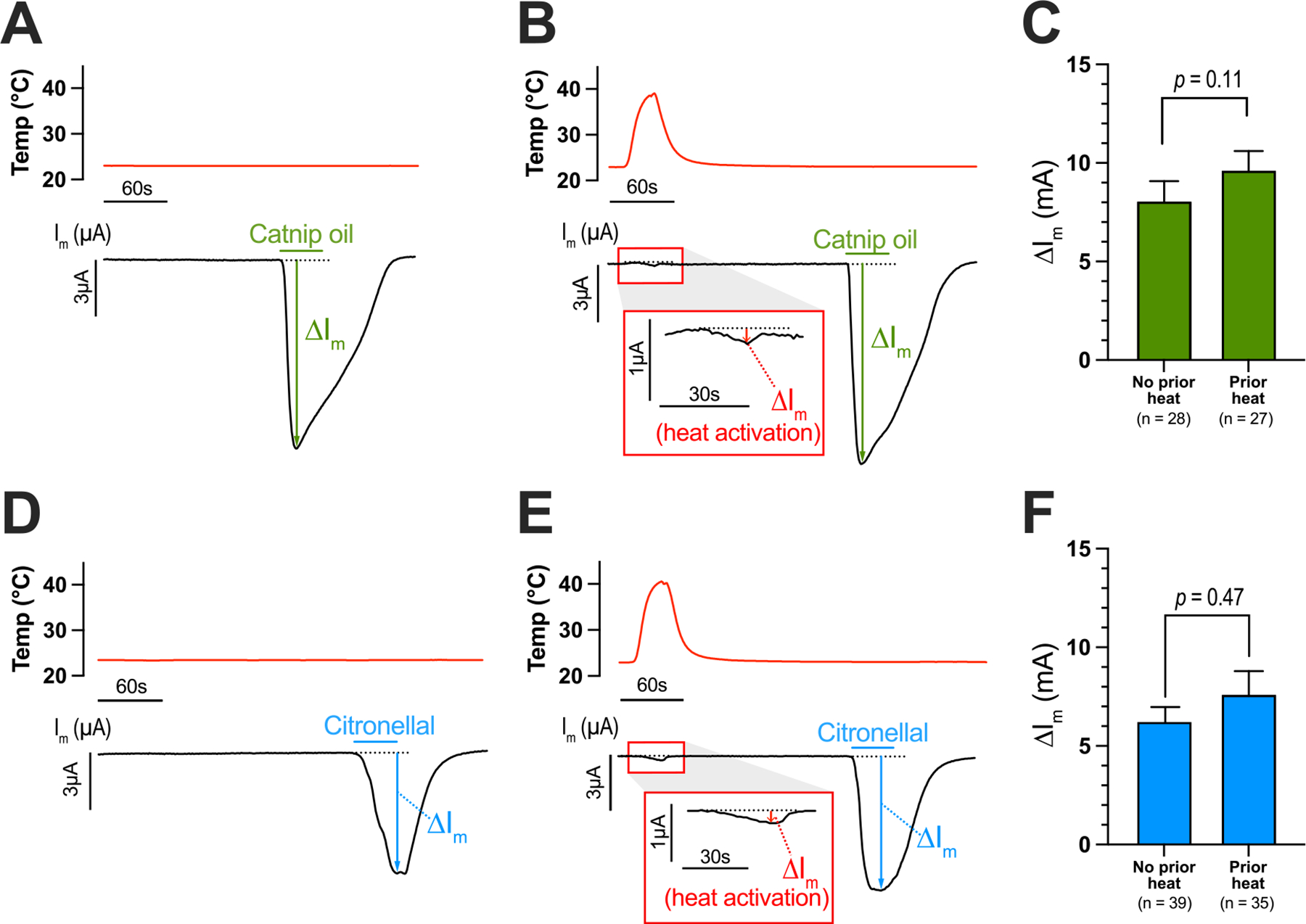
Chemical activation of AaTRPA1 is unaffected by a prior, brief heat-induced activation. Representative traces of bath temperature (red) and I_m_ (black) in AaTRPA1 oocytes exposed to either 0.1 % catnip oil (A&B) or 1 mM citronellal (D&E) at 23 °C without prior heat activation (A&D) or with prior heat activation (B&E). Green and blue horizontal bars indicate delivery of catnip oil or citronellal, respectively, to the oocyte bath. The maximal electrophysiological responses (ΔI_m_) evoked by temperature (red), catnip oil (green), and citronellal (blue) are indicated. Panels C & F compare the mean ΔI_m_ for each agonist (catnip oil in C, citronellal in F) at 23 °C without or with a prior thermal activation. Values are means ± SEM. The *p*-values were determined by unpaired *t*-tests on log-transformed data.

We next determined if the dampened chemical activation of AaTRPA1 by catnip oil or citronellal observed in Fig. 1 and Fig. S5 would also occur at 23 °C after a prior, brief heat activation (third experimental design in section 2.1). First, we re-tested the response of AaTRPA1 oocytes to catnip oil or citronellal at 23 °C without a prior heat activation. Similar to Fig. 1, AaTRPA1 oocytes showed robust, reversible electrophysiological responses of several μA when exposed to catnip oil or citronellal at 23 °C (Fig. 2A&D). In parallel, and in separate oocytes, the bath temperature was briefly elevated to ~38 °C over 40 s before returning it to 23 °C. This heat treatment reversibly activated the channel (see insets of Fig. 2B&E). A few minutes later, the oocytes were treated with catnip oil or citronellal, which elicited ΔI_m_ responses (Fig. 2B&E) similar in magnitude to those observed in oocytes that did not receive the prior heat treatment (Fig. 2A&D). The mean ΔI_m_ in AaTRPA1 oocytes exposed to catnip oil with no prior heat activation (8.09 μA ± 1.09 μA) was similar to that in AaTRPA1 oocytes that received a prior heat activation before exposure to catnip oil (9.66 μA ± 0.94 μA) (Fig. 2C). Likewise, the mean ΔI_m_ in AaTRPA1 oocytes exposed to citronellal with no prior heat activation (6.28 μA ± 0.81 μA) was similar to that in AaTRPA1 oocytes that received a prior heat activation before exposure to citronellal (7.64 μA ± 1.16 μA) (Fig. 2F).

Control oocytes injected with H_2_O exhibited ΔI_m_ responses (0.11 μA ± 0.04 μA, *n* = 11) associated with the brief temperature increase (Fig. S4C&D) that were ~80 % smaller (unpaired *t*-test, *p* = 0.0002) than those observed in AaTRPA1 oocytes (0.57 μA ± 0.07 μA, *n* = 62). Neither catnip oil nor citronellal elicited detectable responses in H_2_O-injected oocytes that were exposed to the brief temperature increase (Fig. S4C&D).

### 3.3. Exposure of Ae. aegypti females to temperatures above the TRPA1 heat activation threshold reduces their avoidance of chemical TRPA1 agonists

**Fig. 3.**
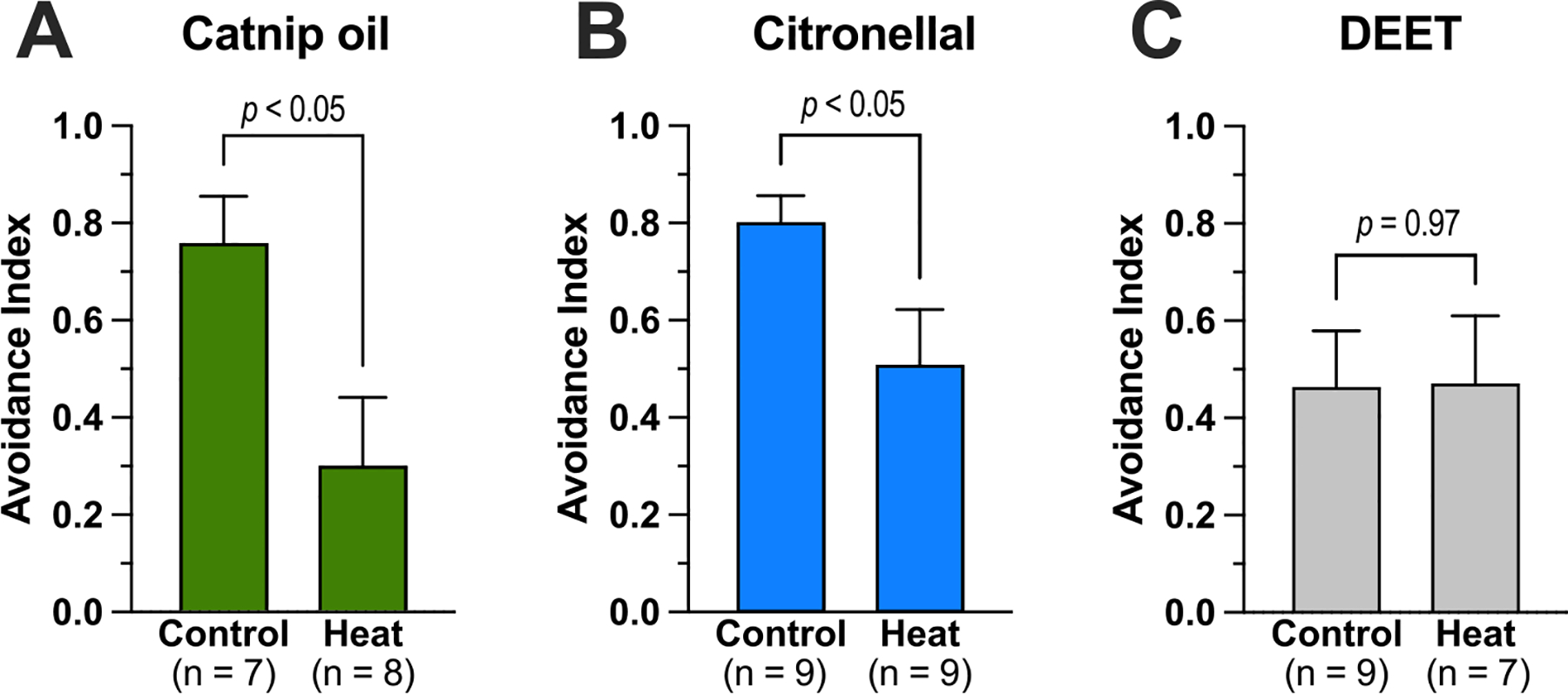
Exposure to heat above the TRPA1 activation threshold reduces the repellency of mosquitoes to catnip oil and citronellal, but not DEET. Avoidance index (AI) of mosquitoes to 0.5 mg catnip oil (A), 1.0 mg citronellal (B), or 0.5 mg DEET (C) during exposure to 26 °C (control) or a heat treatment (see Fig. S2). Values are mean ± SEM. *p*-values were determined by unpaired *t*-tests.

To generate insights into whether exposure of mosquitoes to ambient temperatures above the heat activation threshold of AaTRPA1 impacts their avoidance of catnip oil or citronellal, we performed airborne repellency assays. As shown in Fig. 3A, at 26 °C (control), the mosquitoes strongly avoided catnip oil (AI = 0.76 ± 0.10). In contrast, when mosquitoes were exposed to a heat treatment where the temperature exceeded the thermal activation threshold of AaTRPA1 (see Fig. S2), the mosquitoes were less repelled by catnip oil (Fig. 3A; AI = 0.30 ± 0.14). Similarly, as shown in Fig. 3B, control mosquitoes were more strongly repelled by citronellal (AI = 0.80 ± 0.06) compared to those exposed to the heat treatment (AI = 0.51 ± 0.12). To confirm the above trends were not due to a potentially generic effect of the heat exposure, we repeated the experiments using DEET, a repellent that is not known to agonize insect TRPA1 channels (Shimomura et al., 2022). As shown in Fig. 3C, control mosquitoes appeared to not be as strongly repelled by the less volatile DEET (AI = 0.47 ± 0.12) compared to catnip oil and citronellal. However, mosquitoes exposed to the heat treatment were repelled by DEET (AI = 0.48 ± 0.14) to a similar degree as the control (Fig. 3C). Similarly, the heat treatment did not impact the responses in negative (solvent) controls where 100 % acetone was applied to both filter papers (Fig. S6).

## 4. Discussion

The present study demonstrated a desensitizing effect of heat activation on the responsiveness of an insect TRPA1 channel to chemical agonists. We found that electrophysiological responses of AaTRPA1-expressing oocytes to chemical agonists (i.e., catnip oil, citronellal) were reduced if applied while AaTRPA1 was heat activated. On the other hand, a brief, reversible heat activation of AaTRPA1 prior to treatment with the chemical agonist did not impact the subsequent response. This suggests that continuous heat activation of AaTRPA1 is required to desensitize the channel to chemical agonists. Our results are consistent with those observed in mammalian TRPA1 channels (Wang et al., 2012) where responses to the chemical agonists mustard oil, menthol, and flufenamic acid were all dampened when the channels were heat activated. The similar desensitization to chemical agonists during heat activation in mosquito and mammalian TRPA1 suggests that it might be a widely conserved phenomenon among animal TRPA1 channels.

It is possible that the desensitization of AaTRPA1 to chemical agonists during heat activation is associated with the so-called transient heat activation or heat-induced desensitization that occurs in other arthropod TRPA1 channels (Kohno et al., 2010; Peng et al., 2016; Sato et al., 2014; Viswanath et al., 2003; Wang et al., 2009). That is, while exposed to a prolonged heat stimulus above the thermal activation threshold, arthropod TRPA1 channels show an initial strong activation followed by a reduced response (Kohno et al., 2010; Peng et al., 2016; Sato et al., 2014; Viswanath et al., 2003; Wang et al., 2009). Notably, we observed this phenomenon in AaTRPA1 while it was exposed to continuous heat for 2–3 min (Fig. 1B&D; Fig. S3).

Future studies will be required to elucidate the specific mechanism (s) by which continuous heat activation of AaTRPA1 desensitizes it to chemical agonists, but it may involve inhibition of TRPA1 by intracellular Ca^2+^ and phosphatidylinositol 4,5-bisphosphate, and/or internalization of TRPA1 from the plasma membrane to intracellular vesicles (Talavera et al., 2020). Moreover, continuous heat exposure may impart large conformational changes to the channel that result in reduced accessibility of the chemical agonists to their respective binding sites (Nadezhdin et al., 2021; Wang et al., 2023) and/or inability of TRPA1 to undergo additional conformational changes that would be induced by the binding of a chemical agonist (Sánchez-Moreno et al., 2018). Any of these mechanisms would reduce the number of TRPA1 channels available for chemical activation and lead to a diminished response to chemical agonists as observed in the present study. We also cannot rule out that the desensitization is partially associated with a buildup of intracellular cations (e.g., Ca^2+^ and/or Na^+^) transported into the cells by TRPA1 channels during prolonged temperature activation (e.g., 2–3 min) that could reduce the electrochemical gradient for cation uptake. However, this is unlikely to contribute to the strong desensitization of AaTRPA1 to chemical agonists that we observed within only 30 s of heat activation (Fig. S5).

Our finding of AaTRPA1 desensitization to chemical agonists during heat activation predicts that mosquitoes continuously exposed to ambient temperatures exceeding the thermal activation threshold of AaTRPA1 might be less sensitive to repellents that agonize TRPA1, such as catnip oil or citronellal. Consistent with this prediction, using an airborne repellency bioassay, we found that adult female *Ae. aegypti* exposed to a rapid increase of temperature above the TRPA1 activation threshold of 32 °C were less repelled by catnip oil or citronellal compared to mosquitoes exposed to a temperature below the TRPA1 activation threshold (26 °C). In contrast, mosquitoes were similarly repelled by DEET, which is not known to modulate insect TRPA1 channels (Shimomura et al., 2022), regardless of temperature exposure. Although additional behavioral studies using other bioassays (e.g., arm in cage experiments) will be required to confirm whether this phenomenon is relevant to host-seeking mosquitoes, our results suggest that DEET is a better choice for a repellent (compared to catnip oil, citronellal, and any other natural repellents that may agonize TRPA1) when ambient temperatures exceed the thermal activation threshold of AaTRPA1. This finding may be especially relevant for choosing a repellent for endemic mosquitoes in temperate climates, where ambient temperatures that exceed the mosquito TRPA1 thermal activation threshold are more likely to occur, especially during extreme heat events. For example, the thermal activation threshold of the temperate *Cx. pipiens* is 21.8 °C vs. 32 °C for the tropical *Ae. aegypti* (Li et al., 2019).

In conclusion, the present study adds to a growing field of research that has documented the potential impacts of climate change on mosquito biology and control. For example, global warming may affect mosquito distributions, abundances, growth rates, olfaction, thermal adaptability, responses to pesticides, and arbovirus replication rates (Agyekum et al., 2021; Lahondère et al., 2023; Lahondère and Bonizzoni, 2022; Mack and Attardo, 2024; Reinhold et al., 2018; Rocklöv and Dubrow, 2020). The growing frequency of extreme heat events around the world and rise in average global temperatures (IPCC, 2023; McGregor et al., 2015; NOAA, 2021) motivates further understanding of the potential impacts of these climate phenomena on mosquito biology and control. The present study demonstrates that heat activation of AaTRPA1 desensitizes it to chemical agonists, which may reduce the efficacy of repellents that target AaTRPA1 (e.g., catnip oil, citronellal) when used at ambient temperatures that exceed the thermal activation threshold of AaTRPA1. Thus, the present study provides valuable insights into selecting potentially more effective active ingredients to use for repellent-based mosquito control under the threat of global warming and extreme heat events.

## Supplementary Material

1

## Data Availability

Data will be made available on request.

## Appendix A. Supplementary data

Supplementary data to this article can be found online at https://doi.org/10.1016/j.pestbp.2025.106326.
