# Supplementary material for: Heat activation desensitizes Aedes aegypti transient receptor potential ankyrin 1 (AaTRPA1) to chemical agonists that repel mosquitoes
Source: Pestic Biochem Physiol. Author manuscript; Available in PMC 2026 May 8. (PMC13155204; doi:10.1016/j.pestbp.2025.106326)
Supplement: 1 [file NIHMS2171228-supplement-1.docx]

# Supplemental figures


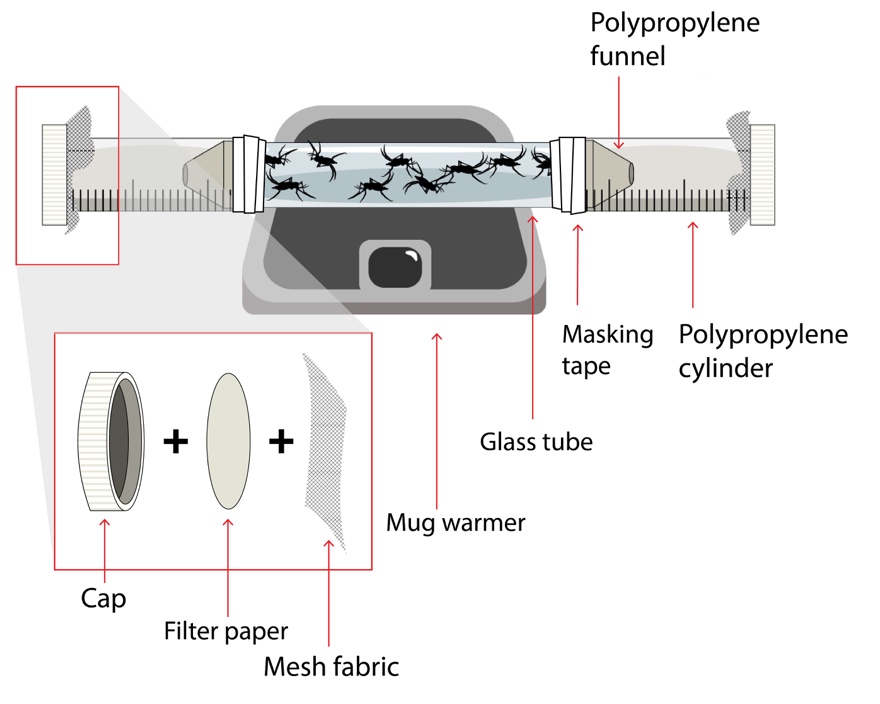


**Fig. S1. Illustration of the mosquito airborne repellency trap bioassay.** See text for details.

**Fig. S2. Temperature change within the glass tube of the mosquito airborne repellency trap bioassay associated with the heat treatment.** Time at which temperature reaches the TRPA1 activation threshold (32 °C) is indicated by dashed red lines. The time and temperature at which at least 80% of mosquitoes have left the glass tube are indicated by the dashed magenta line. Values are means ± SEM; n = 8 independent trials.


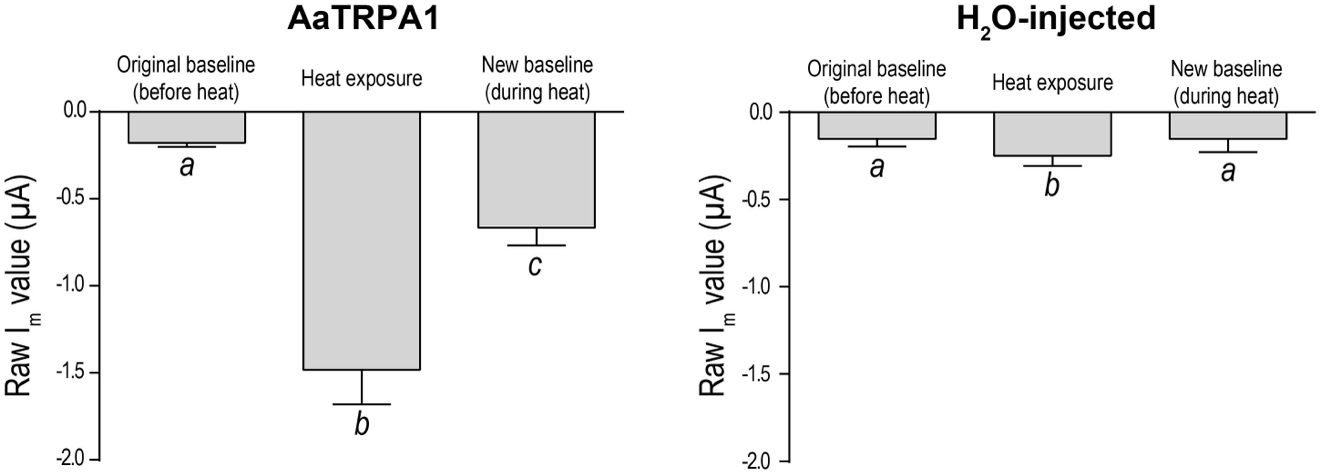


**Fig. S3.** **Effects of continuous heat exposure on baseline I_m_ values in AaTRPA1 and H_2_O-injected oocytes.** Values are raw I_m_ values in voltage-clamped oocytes from the continuous heat treatment experiments of Fig. 1. The ‘original baseline’ is the resting inward I_m_ value immediately prior to heating the bath to ~38°C. The ‘heat exposure’ is the maximum inward I_m_ value reached within the first few min of heat exposure (e.g., heat activation in Fig. 1). The ‘new baseline’ is the resting inward I_m_ value immediately prior to adding the chemical agonist (i.e., catnip oil or citronellal) while the bath is heated at ~38°C. Values are mean ± SEM based on measurements in 60 AaTRPA1 oocytes and 10 H_2_O-injected oocytes. Lower-case italicized letters indicate statistical categorization of the means within AaTRPA1 or H_2_O-injected oocytes as determined with a one-way repeated measures ANOVA and Tukey’s multiple comparisons test (*P* < 0.05). Note that in AaTRPA1 oocytes the new baseline is significantly greater than that of the original baseline, but lower than that of the maximal heat-induced response, suggesting the oocytes maintain an activated state during heat exposure albeit in a less activated state than the initial heat exposure. In H_2_O-injected oocytes, the new baseline during exposure is similar to that of the original baseline. The initial heat exposure elicits a small, but significant, change in I_m_ that fully dissipates during heat exposure.


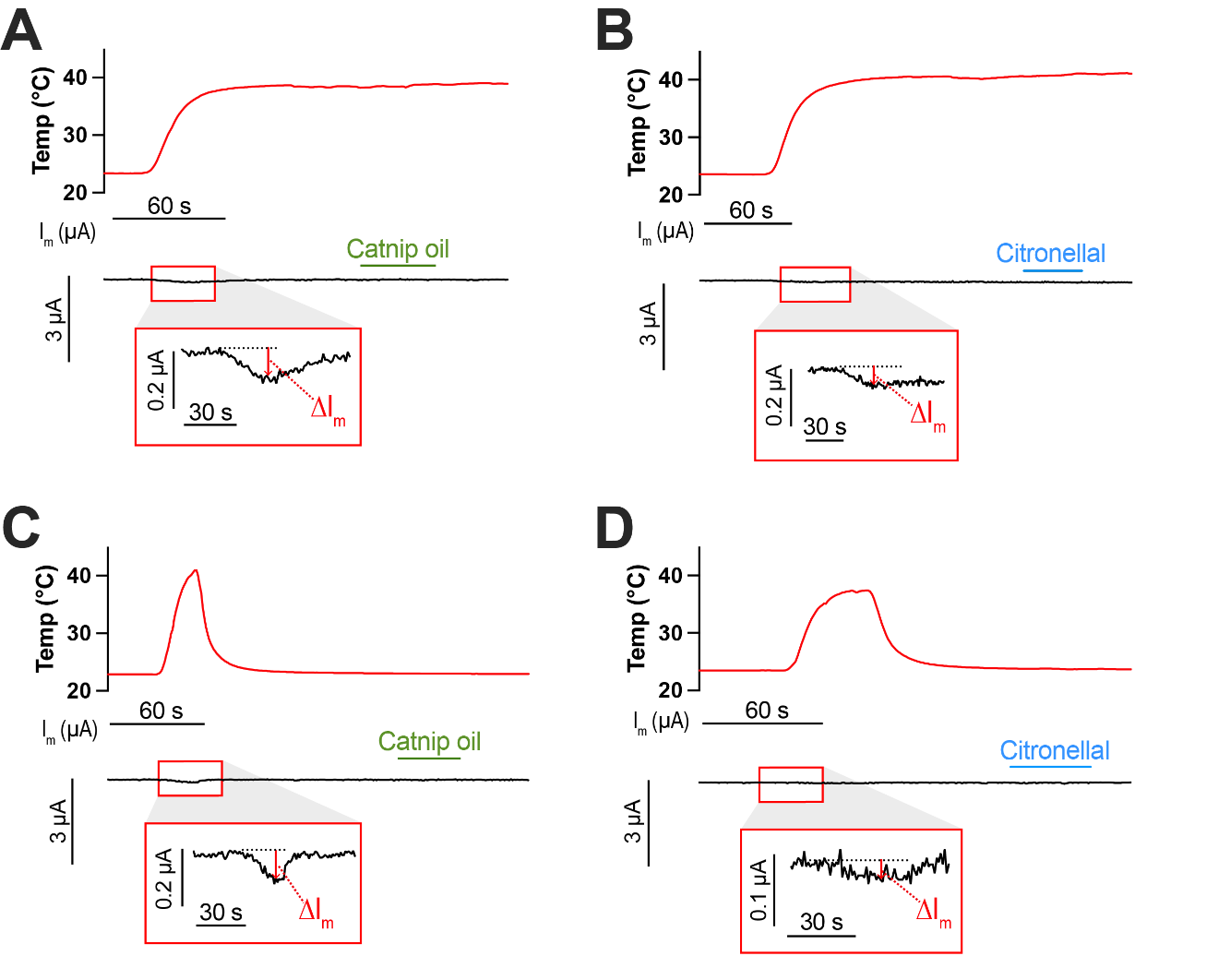


Fig. S4. Representative traces of bath temperature (red) and I_m_ (black) in oocytes injected with nuclease-free H_2_O during brief (A&C) and continuous (B&D) heat exposure.


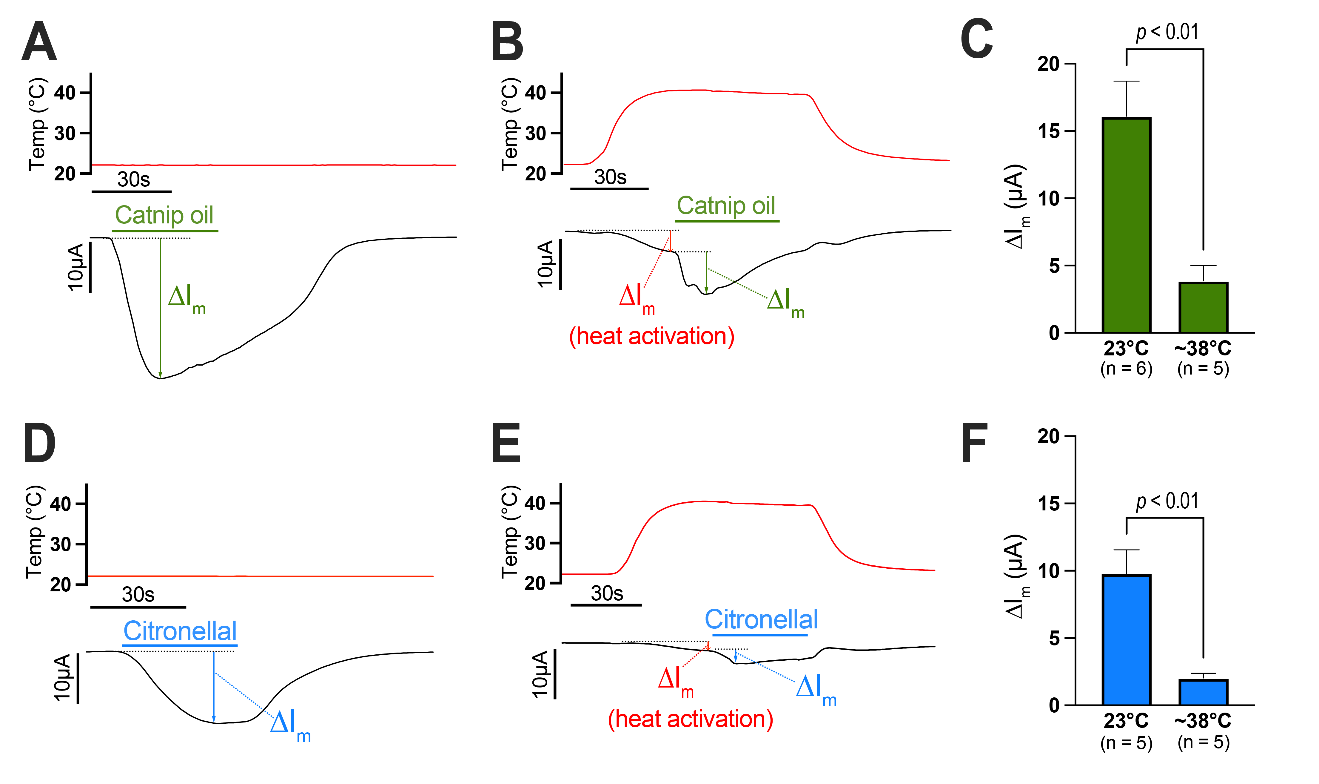


**Fig. S5. Chemical activation of AaTRPA1 is dampened by a brief, continuous heat activation.** Representative traces of bath temperature (red) and I_m_ (black) in AaTRPA1 oocytes exposed to either 0.1% catnip oil (A&B) or 1 mM citronellal (D&E) at 23 °C (A&D) or ~38 °C (B&E). Green and blue horizontal bars indicate exposure of oocytes to catnip oil or citronellal, respectively. The maximal electrophysiological responses (ΔI_m_) evoked by temperature (red), catnip oil (green), and citronellal (blue) are indicated. Panels C & F compare the mean ΔI_m_ for each agonist (catnip oil in C, citronellal in F) at 23 °C vs. ~38 °C. Values are means ± SEM. The *p*-values were determined by either an unpaired Mann-Whitney test (C) or an unpaired *t*-test (F). The mean ΔI_m_ in AaTRPA1 oocytes exposed to catnip oil at 23 °C (16.08 µA ± 2.62 µA) was ~4-fold larger than that in heat-activated AaTRPA1 oocytes exposed to catnip oil at ~38 °C (3.86 µA ± 1.16 µA). Similarly, the mean ΔI_m_ in AaTRPA1 oocytes exposed to citronellal at 23 °C (9.80 µA ± 1.77 µA) was ~5-times larger than that in heat-activated AaTRPA1 oocytes exposed to citronellal at ~38 °C (2.00 µA ± 0.37 µA).


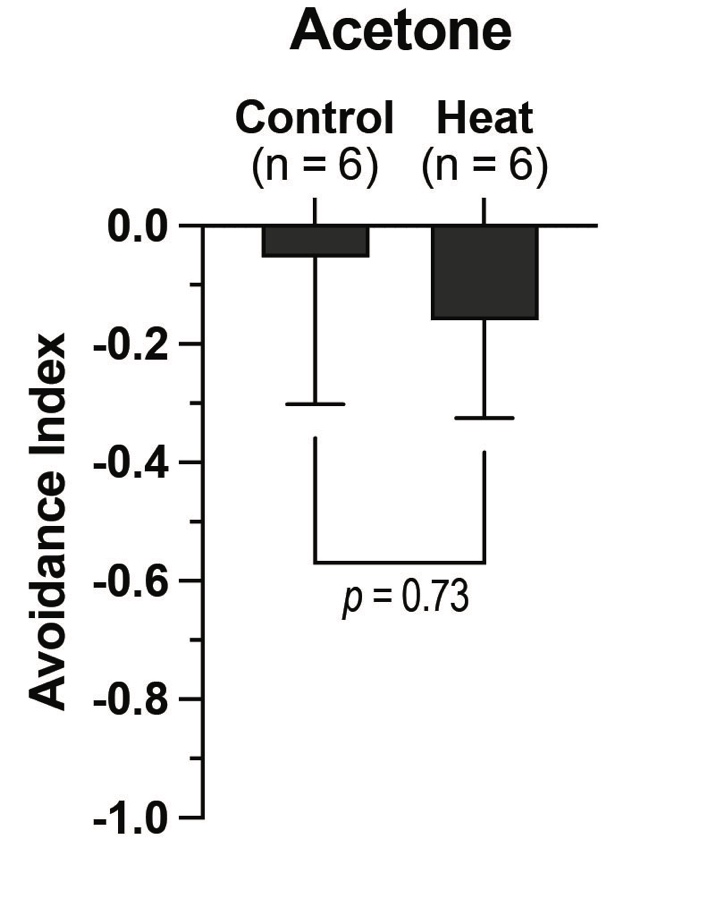


Fig. S6. Mean avoidance index of mosquitoes in airborne repellency assays for negative (solvent) controls where 100% acetone is applied to both filter papers. Values are mean ± SEM. *p*-value was determined by an unpaired *t*-test.
